# Genome-Wide Analysis of Heteroduplex DNA in Mismatch Repair–Deficient Yeast Cells Reveals Novel Properties of Meiotic Recombination Pathways

**DOI:** 10.1371/journal.pgen.1002305

**Published:** 2011-09-29

**Authors:** Emmanuelle Martini, Valérie Borde, Matthieu Legendre, Stéphane Audic, Béatrice Regnault, Guillaume Soubigou, Bernard Dujon, Bertrand Llorente

**Affiliations:** 1 CEA DSV/IRCM, Unité Mixte de Recherche 217 Radiobiologie Moléculaire et Cellulaire, Centre National de la Recherche Scientifique, Commissariat à l'Energie Atomique et aux Energies Alternatives, Fontenay aux Roses, France; 2 Unité Mixte de Recherche 218, Centre National de la Recherche Scientifique, Paris, France; 3 Centre de Recherche, Institut Curie, Paris, France; 4 Unité Propre de recherche 2589, Structural and Genomic Information Laboratory, Centre National de la Recherche Scientifique, Mediterranean Institute of Microbiology IFR88, Aix-Marseille University, Parc Scientifique de Luminy, Marseille, France; 5 UMR 7144, Adaptation et Diversité en Milieu Marin, Equipe Evolution du Plancton et Paléo-Océans, Station Biologique de Roscoff, Centre National de la Recherche Scientifique and University Pierre and Marie Curie-Paris, Roscoff, France; 6 Génopole, Institut Pasteur, Paris, France; 7 Unité de Génétique Moléculaire des Levures, Institut Pasteur, Centre National de la Recherche Scientifique/University Pierre and Marie Curie-Paris, Paris, France; 8 Unité Propre de Recherche 3081, Laboratory of Genome Instability and Carcinogenesis, conventionné par l'Université d'Aix-Marseille 2, Centre National de la Recherche Scientifique, Marseille, France; National Cancer Institute, United States of America

## Abstract

Meiotic DNA double-strand breaks (DSBs) initiate crossover (CO) recombination, which is necessary for accurate chromosome segregation, but DSBs may also repair as non-crossovers (NCOs). Multiple recombination pathways with specific intermediates are expected to lead to COs and NCOs. We revisited the mechanisms of meiotic DSB repair and the regulation of CO formation, by conducting a genome-wide analysis of strand-transfer intermediates associated with recombination events. We performed this analysis in a SK1 × S288C *Saccharomyces cerevisiae* hybrid lacking the mismatch repair (MMR) protein Msh2, to allow efficient detection of heteroduplex DNAs (hDNAs). First, we observed that the anti-recombinogenic activity of MMR is responsible for a 20% drop in CO number, suggesting that in MMR–proficient cells some DSBs are repaired using the sister chromatid as a template when polymorphisms are present. Second, we observed that a large fraction of NCOs were associated with trans–hDNA tracts constrained to a single chromatid. This unexpected finding is compatible with dissolution of double Holliday junctions (dHJs) during repair, and it suggests the existence of a novel control point for CO formation at the level of the dHJ intermediate, in addition to the previously described control point before the dHJ formation step. Finally, we observed that COs are associated with complex hDNA patterns, confirming that the canonical double-strand break repair model is not sufficient to explain the formation of most COs. We propose that multiple factors contribute to the complexity of recombination intermediates. These factors include repair of nicks and double-stranded gaps, template switches between non-sister and sister chromatids, and HJ branch migration. Finally, the good correlation between the strand transfer properties observed in the absence of and in the presence of Msh2 suggests that the intermediates detected in the absence of Msh2 reflect normal intermediates.

## Author Summary

Sexual reproduction consists in fusing two complementary gametes carrying only one set of chromosomes (haploids) to form a cell with two sets of homologous chromosomes (diploid). Gametes are generated through meiosis, a specialized cell division occurring in diploid organisms. For proper meiotic division to occur, homologous chromosomes need physical connections acquired through recombination that exchange chromosome arms (crossovers) and thereby contribute to genetic diversity. Recombination is induced by numerous chromosome breakages, but only a subset yields crossover recombinants, the remaining yielding non-crossover recombinants. Control of crossover formation is poorly understood. For this reason, precise knowledge of the meiotic recombination mechanisms is essential. Current models are based on studies performed at a few loci in model organisms. We revisited these models using an original approach that allowed us to study the DNA scars left at all chromosome breakage sites during single meioses in baker's yeast. We found that crossover formation is more dynamic than anticipated, which led us to propose variations of current crossover formation models. We also revealed that a significant fraction of non-crossovers do not arise from the canonical pathway, raising the possibility of a common pathway with crossover formation.

## Introduction

Meiotic crossovers (COs) are reciprocal exchanges of chromosome arms between homologous chromosomes (homologs). They generate genetic diversity and establish physical links between homologs. In many organisms COs are crucial for accurate homolog segregation at meiotic division I, and the absence of COs leads to mis-segregation of homologs and aneuploid gametes (for review [1]). Crossover control is therefore of extreme importance for normal meiosis.

Crossovers result from the repair of programmed meiotic DNA double-strand breaks (DSBs) by the homologous recombination machinery. In most organisms DSBs outnumber COs, although to various degrees. A subset of DSBs that do not give COs is repaired without reciprocal exchange of chromosome arms and gives non-crossover products (NCOs) that can only be identified by gene conversions associated with the recombination process. DSB formation involves several proteins including the topoisomerase-like transesterase Spo11 protein that harbors the nucleolytic activity [2]–[4].

After DSB formation and Spo11 removal from the 5′ ends of the breaks [5], 3′ single-stranded tails are generated and initiate recombination with homologous sequences [6] to ultimately produce COs and NCOs. Genetic and physical analyses performed in *Saccharomyces cerevisiae* suggest that the decision to form either a CO or a NCO is made before or during the transition between DSB formation and strand invasion of the homolog by one end of the DSB [7]-[10]. The molecular nature of this decision point remains to be elucidated.

Two major pathways are involved in meiotic CO formation [11], [12]. The ZMM pathway depends on the synaptonemal complex proteins Zip1, Zip2, Zip3, the Mer3 helicase and the Msh4/Msh5 proteins, homologs of the bacterial mismatch repair protein MutS [9], [13]. This pathway relies on the integrity of the synaptonemal complex [14], a highly conserved structure that connects the homologs axes over their entire length (for review [15]). The Mus81 pathway depends on the nuclease activity of Mus81 to resolve recombination intermediates [16], [17], independently of the synaptonemal complex integrity [12], [18]. Residual COs in *S. cerevisiae* strains lacking both pathways suggest the existence of a third pathway that is likely repressed in a wild type context [11]. The balance between these pathways varies among organisms. CO formation results from both the ZMM and Mus81 pathways in *S. cerevisiae*, *Arabidopsis thaliana* and mammals, whereas it mainly results from the Mus81 pathway in *Schizosaccharomyces pombe* and from the ZMM pathway in *Caenorhabditis elegans*.

The molecular mechanisms involved in CO control are relatively unknown despite several levels of CO regulation (for review [19]). In organisms where the ZMM pathway is present, COs show interference, in that formation of a CO inhibits CO formation nearby. In addition, in genetic backgrounds where the number of DSBs is reduced, COs tend to be maintained at the expense of NCOs but the molecular mechanism of this crossover homeostasis remains unknown [20]. Both CO interference and CO homeostasis participate in the non-random distribution of COs along and among chromosomes.

**Figure 1:**
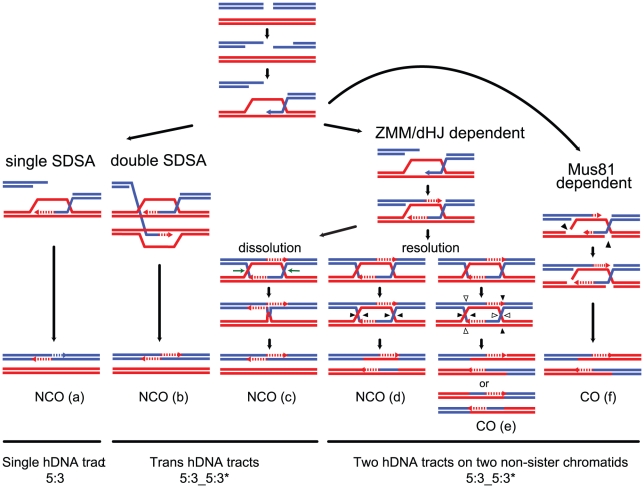
Strand transfers during canonical meiotic DSB repair pathways. For simplicity, only two homologous DNA molecules are represented, one red and one blue. The 3′ DNA end is identified by an arrow when appropriate. Newly synthesized DNA is represented as a dotted line. After a DSB is made, 5′ to 3′ end resection followed by 3′ end invasion of an intact homoduplex DNA molecule are common steps to all recombination pathways. Three major recombination pathways are distinguished according to the intermediates formed. On the left, after 3′ end invasion, DNA synthesis extends the invading end prior to the dismantling of this evanescent intermediate. The DSB is eventually repaired after annealing of the two ends, and gap fill in (a) (SDSA model, [24]). This pathway generates exclusively NCOs with only one hDNA tract. Both ends of a DSB can formally engage in two independent SDSA reactions, generating also a NCO but with two hDNA tracts distributed on the same chromatid in a trans configuration (b). In the middle, after 3′ end invasion, a stable SEI intermediate is formed and is processed into a double Holliday junction-containing intermediate. dHJ resolution can formally lead to CO if the four nicks (arrowheads) cleave four different DNA strands (e), or NCO if the four nicks affect only two DNA strands (d) (these two pathways illustrate the canonical DSBR model [21]). Note that filled and unfilled arrowheads illustrate the two possible resolutions of the same dHJ. The crossover point is defined by the two vertical arrowheads. The top CO pattern therefore corresponds to resolution by the filled arrowheads, and the bottom CO pattern corresponds to resolution by the unfilled arrowheads. Both COs and NCOs resulting from dHJ resolution present two hDNA tracts distributed on the two non-sister chromatids involved in the repair reaction. Alternatively, a dHJ can be dissolved by the combined action of a helicase and a type I topoisomerase and give rise to a NCO with two hDNA tracts on the same chromatid in a trans configuration (c). On the right, after 3′ end invasion, a less stable intermediate than the aforementioned SEI is formed and contains two nicked HJs (f). Structure-specific endonucleases like Mus81 can process such an intermediate into a CO with two hDNA tracts distributed on the two non-sister chromatids involved in the repair reaction, as for the canonical DSBR model (f). The notation 5:3_5:3* stands for two consecutive half conversions or hDNA tracts with the same global strand asymmetry but with different strand distributions. Trans hDNAs are defined by two such hDNA tracts on the same chromatid, while dHJ resolution leads to two such hDNA tracts on two non-sister chromatids.

The original DSB repair model [21] proposed that both COs and NCOs result from distinct resolutions of a common recombination intermediate containing a double Holliday junction (dHJ) (Figure 1). However, subsequent physical and genetic analyses at a few recombination hot spots in *S. cerevisiae* showed that meiotic dHJs are almost exclusively resolved as COs, which depend on the integrity of the ZMM proteins [7], [9], [10]. This implies that NCOs result from an alternative recombination pathway, such as synthesis-dependent strand annealing (SDSA), which does not produce dHJs [22]–[24]. The SDSA pathway is characterized by more evanescent strand invasion intermediates than the long-lived intermediates leading to dHJs [7], [10]. In parallel, however, genetic studies led to the proposal that part of NCOs could also come from dissolution of dHJs [25], a reaction known to be catalyzed *in vitro* by the combined action of a RecQ helicase and a type I topoisomerase [26], [27].

Meiotic recombination is frequently associated with non-Mendelian segregation or 3:1 segregation of genetic markers. Less often, meiotic recombination is associated with post meiotic segregation (PMS) of genetic markers, which is identified by the formation of sectored colonies in fungi. Early studies based on these observations led to the original models of meiotic recombination. Holliday [28] as well as Meselson and Radding [29] suggested that heteroduplex DNA, which can lead to PMS, was the basic intermediate of recombination. Depending on the way mismatches are repaired, genetic markers within hDNA can be restored or converted yielding 4∶4 or 3∶1 segregation patterns, respectively. On another hand, Szostak et al. [21] favored the formation of double-strand gaps to explain the high frequency of 3∶1 segregation patterns. Extensive studies of PMS and the identification of mutants that increase PMS performed by Fogel and colleagues confirmed the formation of hDNA during meiotic recombination [30]–[32]. The association of hDNA with recombination intermediates was confirmed more recently by physical analysis [33]. Such mismatches are normally recognized and repaired by the mismatch repair machinery (MMR) (for review [34]). The configuration of hDNA tracts is expected to vary depending on the recombination pathway and the specificity of the DNA transactions taking place (Figure 1) (for review [35]). Previous studies identified hDNAs at a few DSB hot spots either by using poorly repairable hairpin/loop extruding palindromes or by inactivating MMR. They revealed complex hDNA patterns and therefore proposed variations of the canonical recombination models [25], [36], [37].

In the presence of MMR, DNA polymorphism is a barrier to recombination and may lead to meiotic sterile progeny and therefore reproductive isolation of populations. In bacteria, this recombination barrier has been observed during transformation with polymorphic DNA [38], [39] as well as during conjugation between diverged species [40]–[42]. In *S. cerevisiae* the efficiency of hDNA formation both in mitotic and meiotic cells decreases with increasing sequence divergence [43], [44]. In eukaryotes, the current model proposes that homologs of the MutS bacterial MMR protein sense and reject mispaired hDNA intermediates formed during 3′ end invasion of the donor sequence. This hypothesis is reinforced by the enrichment in the MutS homolog Msh2 at the donor and recipient sequences near a DSB in the presence of sequence polymorphism (for review [34]).

Until recently most studies of meiotic COs, NCOs and hDNAs were based on a few loci corresponding to DSB hot spots and required the introduction of genetic markers. It is possible that the features and the balance between recombination pathways taking place at these loci do not reflect the average behavior of all loci. DNA arrays as well as deep sequencing allow the use of the natural polymorphic sites between diverged strains as markers to identify all recombination events between homologs generated during a single meiosis [45]–[48]. In order to better understand meiotic DSB repair mechanisms on a genome-wide level and therefore to explore CO formation control, we studied the DNA strand composition of the products of virtually all the interhomolog meiotic recombination events from two individual meioses of a SK1 x S288C hybrid lacking the Msh2 protein, using Affymetrix DNA tiling-arrays. This study provides for the first time a genome-wide view of hDNAs associated with COs and NCOs. This large data set allows a reassessment of current meiotic recombination models.

## Results/Discussion

### Rationale for the identification of the global landscape of COs, NCOs, and hDNAs in a SK1 × S288C hybrid using segregation of natural polymorphisms

**Figure 2:**
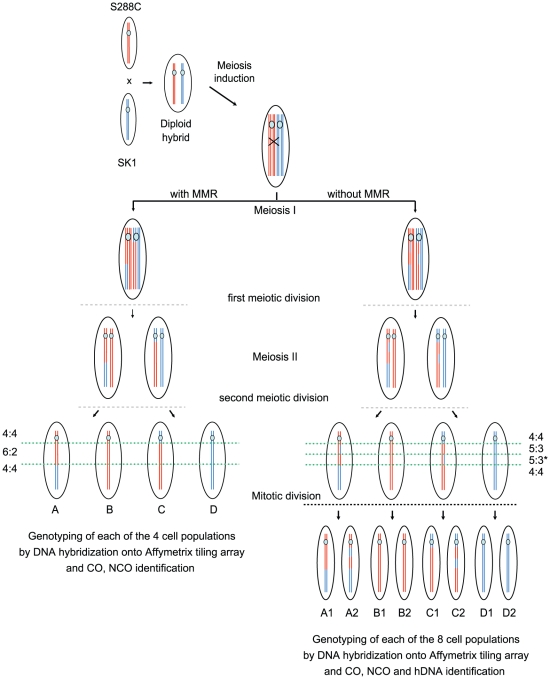
Rationale for the identification of the global landscape of COs, NCOs, and hDNAs. Only one DNA molecule of the S288C (red) and SK1 (blue) strains are represented for simplicity. After induction of meiosis, Spo11 generates a DSB on the SK1 chromosome that is repaired by copying genetic information from the S288C chromosome. This homologous recombination process can give rise to either a NCO or a CO as represented here (black cross) and generates hDNA tracts (DNA segments containing two strands of different parental origins i.e. one SK1 (blue) and one S288C (red) strand here). In the presence of MMR (left part), mismatches present in hDNAs are repaired toward either restorations or full conversions leading to 4∶4 and 6∶2 segregation patterns respectively of the corresponding markers in the hybrid progeny. A full conversion is represented here. In the absence of MMR (right part), mismatches from hDNAs are left unrepaired and the corresponding markers present a 5∶3 segregation pattern as shown here. The asterisk indicates that distinct hDNA tracts generate the same global 5∶3 segregation of nearby markers. hDNAs are homogenized into homoduplex DNAs at the first mitotic division of the spores. Genotyping by DNA hybridization onto Affymetrix tiling arrays of the four (presence of MMR) or eight (absence of MMR) clonal cell populations allows CO and NCO identification from recombination products. hDNA identification is possible only in the absence of MMR by genotyping of the eight populations.

In order to identify meiotic COs and NCOs genome wide, we used an approach derived from the original work of Winzeler et al. [48]. It consists of crossing two polymorphic isolates that give rise to a fertile hybrid, in our case SK1 and S288C, inducing meiosis and genotyping each cell population coming from the four spores of a given meiosis to identify recombination events. For genotyping, each DNA from the four cell populations was hybridized onto one Affymetrix DNA tiling array (GeneChip S. cerevisiae Tiling 1.0 R Array) containing the genome of the S288C parental strain. The hybridization profiles were compared to those from the two parental strains to reveal their origins [49]. Such a strategy allows the identification of virtually all the single nucleotide polymorphisms between the parental strains [45], [46], [48], [50] (Figure 2, Figures S1 and S2).

One major difference between our study and previous similar genome-wide studies is the hybrid used. We crossed SK1 and S288C that have about 0.7% sequence divergence [51], [52], which is more than between YJM789 and S288C (0.5%, [51], [53]) previously used [45], [46]. Nevertheless, our hybrid goes through meiosis efficiently and spore viability is 70% in the presence of MMR and 63% in the absence of Msh2. Importantly, the about 62000 sequence polymorphisms between SK1 and S288C that we used as markers are homogenously distributed along the genome, with no large region completely devoid of polymorphism [51], [54]. This gives an average marker density of 1 per 194 nucleotides, with 97.5% of the inter-marker distances smaller than 1000 nucleotides and a median inter-marker distance of 77 bp. Only 11 regions without markers are longer than 10 kb and most of them correspond to Ty-containing loci.

COs and NCOs were identified after analysis of the segregation patterns of all the natural polymorphic sites in the meiotic progeny of the SK1 x S288C *S. cerevisiae* hybrid. In the absence of recombination, all markers show a unique and continuous Mendelian segregation profile (2∶2). COs, which characterize reciprocal exchanges between chromosome arms, lie at the junction between consecutive regions with two different Mendelian segregations of markers. Non-Mendelian segregation of markers (3∶1) at the exchange point reflects the presence of a gene conversion associated with the CO. When markers present a non-Mendelian segregation without chromosome arm exchange, they characterize a NCO.

DSB repair by homologous recombination generates hDNA tracts. The patterns of hDNA tracts are expected to vary upon repair pathways. Analysis of hDNA patterns therefore provides a powerful tool to decipher recombination pathways [36], [55]. Under normal circumstances, mismatches within hDNA are repaired by the MMR machinery. Inactivation of MMR is a common genetic tool to reveal hDNA intermediates. In our case, we chose to disrupt *MSH2* to inactivate MMR and reveal hDNA intermediates, because Msh2 recognizes a large spectrum of mismatches but does not affect meiotic recombination *per se* in the absence of DNA polymorphism, unlike Mlh1 [56]. In the absence of MMR, haploid spores give rise to “mixed colonies” with information from both parents at each polymorphic hDNA. To reveal and trace hDNAs produced in a single meiosis, we therefore separated the mother cell from the daughter cell formed after the first mitotic division of each spore and genotyped the eight resulting cell populations from two meioses (Figure 2). This approach provides the genetic identity of each of the eight DNA strands from the four chromatids of the diploid hybrid after meiotic recombination (Figure S1 and S2) [57]. In the absence of recombination, markers show a continuous Mendelian segregation profile (4∶4). COs lie in between two consecutive regions with different Mendelian segregations. Gene conversions are characterized by either half- or full conversions showing respectively a 5∶3 or 6∶2 segregation profile of the markers. hDNAs correspond to half-conversions and are characterized by a 5∶3 segregation profile and can be associated with both COs and NCOs.

### MMR reduces COs genome-wide in a SK1 × S288C polymorphic hybrid, which suggests an increase of DSB repair using the sister chromatid

**Figure 3:**
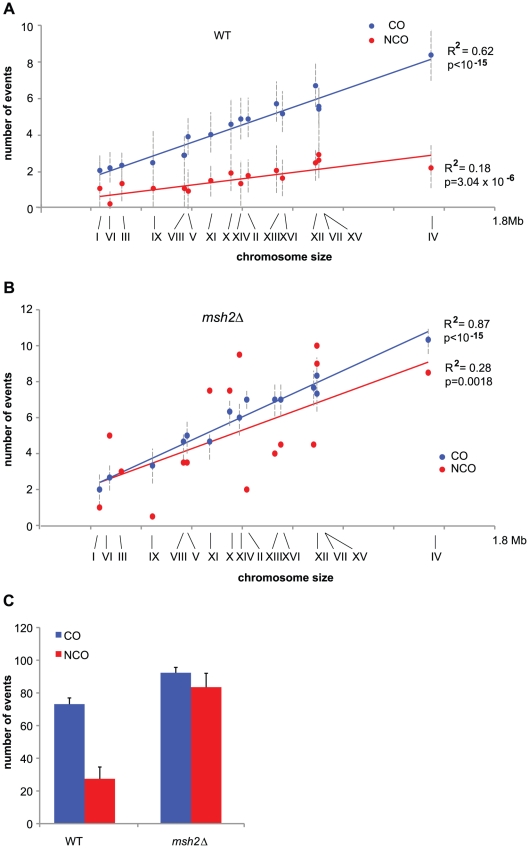
COs and NCOs in a SK1 × S288C hybrid in the presence and absence of MMR. (A) Mean values of COs (blue dots) and NCOs (red dots) per chromosome out of 7 wild type meioses are represented as a function of chromosome size. Chromosome numbers are indicated under the x axis. Vertical dotted lines represent standard deviations. Linear regression curves are represented, with R^2^ corresponding to the square of Pearson's product-moment correlation coefficient, and p being the probability that R^2^ is equal to zero. (B) Same as in A but for a *msh2Δ* hybrid. Note that COs data come from three meioses and NCOs data come from two meioses. (C) Mean values of COs (blue bars) and NCOs (red bars) for wild type and *msh2Δ* hybrids with standard deviations indicated except for *msh2Δ* NCOs where the deviation to the mean has been indicated. Both CO and NCO numbers are significantly higher in *msh2Δ* compared to wild type (p<0.05, Wilcoxon test).

We mapped COs in seven meioses from a *S. cerevisiae* hybrid obtained by crossing a wild type SK1 isolate and a wild type S288C isolate and in three meioses from a similar hybrid missing both alleles of *MSH2*. We identified 73 and 92 COs per meiosis on average in the presence and absence of Msh2, respectively (Figure 3). All chromosomes received at least one CO and the distribution of CO per chromosome is positively correlated to chromosome size (Figure 3A and 3B), confirming previous observations in a YJM789 x S288C hybrid [45], [46]. The correlation between CO number and chromosome size is stronger in the absence of Msh2 (compare R^2^ in Figure 3A and 3B), suggesting that the presence of polymorphisms slightly affects CO distribution. The significant increase in COs (p = 0.021, Wilcoxon test) in the absence of Msh2 is spread over the entire genome but the median distance between two COs is not significantly different from the 123 kb observed in a wild type hybrid (p = 0.18, Wilcoxon test, data not shown). The fact that spore viability is not improved in the absence of Msh2 despite an increase in properly distributed COs may result at least in part from the accumulation of recessive lethal mutations during the vegetative growth of the hybrid. Because Msh2 does not impact CO level in the absence of polymorphism [58], our results show that the polymorphism between SK1 and S288C leads to a 20% drop in COs genome-wide through the action of Msh2. This suggests that some DSBs are repaired using the sister instead of the non-sister chromatid. Such an hypothesis is supported by the recent finding that a meiotic DSB formed at a locus lacking direct homology on the homolog is efficiently repaired with the sister chromatid [59]. Alternatively, we cannot exclude that Msh2 shuttles potential CO intermediates into an interhomolog NCO path with limited strand transfer, the size of which would be below our detection threshold.

Surprisingly, the 92 COs per meiosis on average in a SK1 x S288C hybrid lacking Msh2 are similar to the 90-95 COs observed in a YJM789 x S288C wild type hybrid [45], [46] and to the about 86 COs determined genetically in homozygous *S. cerevisiae* isolates [60], [45]. The results obtained with the two hybrids in the presence of Msh2 look contradictory since a recombination barrier imposed by the MMR seems to exist only in the SK1 x S288C hybrid but not in the YJM789 x S288C hybrid despite a significant level of sequence polymorphism. We envision several possible explanations. (i) The number of COs, as genetically determined in homozygous *S. cerevisiae* isolates, may be underestimated. In such a case, we would expect an increase in COs in the absence of functional MMR in a YJM789 x S288C, which has not been tested. (ii) MMR could be partially defective in the YJM789 x S288C, retaining its ability to repair mismatches necessary for gene conversions, but having lost its anti-recombinogenic activity. (iii) Only the sequence polymorphism between SK1 and S288C is above the threshold that triggers the anti-recombinogenic activity of the MMR. (iv) Finally, it is also possible that COs tend to be limited and maintained around 90 by the meiotic *S. cerevisiae* program. In this case, the MMR anti-recombinogenic action would be masked by CO limitation in the YJM789 x S288C background, but not in the SK1 x S288C hybrid, where sequence polymorphism becomes too high.

### MMR reduces NCOs genome-wide in a SK1 × S288C polymorphic hybrid

Out of seven wild type meioses, we identified 27 NCOs per meiosis on average. In the absence of Msh2, we identified 77 and 92 hDNA patterns associated with NCOs in two meioses with 88 and 93 COs, respectively (Figure 3C). We did not analyze NCOs in the third *msh2Δ* meiosis that was used for CO analysis because of technical problems. As for COs, the number of NCOs is positively correlated to chromosome size but this correlation is weaker due to a higher variability of events per chromosome (Figure 3A and 3B). Local lack of markers, short conversion tracts, and restoration, can all lead to an underestimation of strand transfer events [46]. If this fraction of events is similar for COs and NCOs, an assumption that may not be correct, it would be reflected by the fraction of COs where no strand transfers have been detected, i.e. 14% in the absence of MMR and 23% in the presence of MMR. Under this assumption, the actual average NCOs number per meiosis would be 98 in the absence of MMR and 35 in the presence of MMR. Overall, the number of NCOs detected in the absence of Msh2 was about 3 times higher than the number of NCOs observed in the presence of Msh2 (Figure 3C). Interestingly, in the absence of Msh2, CO and NCO numbers per meiosis approached parity (92 and 98 on average, respectively) and their sum is compatible with the lowest estimates of 140-170 DSBs per meiosis [61] and 160 DSBs per meiosis [62]. This suggests that the low level of NCOs observed in a wild type context mainly results from a Msh2-related activity.

MMR can mask NCOs by preventing recombination using a homologous chromatid and triggering repair using a sister chromatid as observed for COs (see above). MMR can also mask NCOs by restoring parental information, an idea supported by the higher fraction of COs associated with strand transfer in the absence of Msh2 compared to a wild type context (86% versus 77%). Assuming there is no MMR bias toward either conversion or restoration at NCO sites, we would expect roughly as many conversions and restorations associated with NCOs. This would make the number of recombination events we observed in a wild type context compatible with an estimate of 140-170 DSBs per meiosis [61], [62] without involving repair from the sister chromatid. However, in case there is a bias toward conversion at NCO sites as observed in [63], then the number of recombination events we observed would be compatible with repair using the sister chromatid or repair with the homolog but without detectable conversions.

Finally, given the partial defect in mismatch repair due to negative epistasis between the *MLH1* and *PMS1* genes from SK1 and S288C [64] it is possible that we missed some conversion events in the presence of Msh2. Since the fraction of CO associated with a conversion event that we observed with the SK1 x S288C hybrid is comparable with the one from studies using the YJM789 x S288C hybrid, we anticipate that the number of missed NCOs due to the partial MMR defect is negligible.

### Unrepaired hDNAs in the absence of MMR reveal two major NCOs classes

Formally, NCOs may arise from the SDSA pathway as well as the processing of dHJs. Simple SDSA, which consists in the invasion of a homologous sequence by a single end, generates a single 5∶3 hDNA tract [22], [24]. SDSA has been shown to occur during meiosis [23]. Although not formally demonstrated, the two ends of a single DSB could engage SDSA independently and generate two 5∶3 tracts in trans configuration on the same chromatid (double SDSA pathway). Such trans hDNA pattern could also arise from the dissolution of a dHJ by the combined action of a helicase and a topoisomerase I, like Sgs1 and Top3 as proposed by Gilbertson et al. [25]. The resolution of dHJs could also generate NCOs and leave two adjacent 5∶3 hDNA tracts onto the two recombining non-sister chromatids. Current models favor the idea that NCOs are mainly formed by the SDSA pathway [7], [23] and that dHJs mainly give rise to COs.

**Figure 4:**
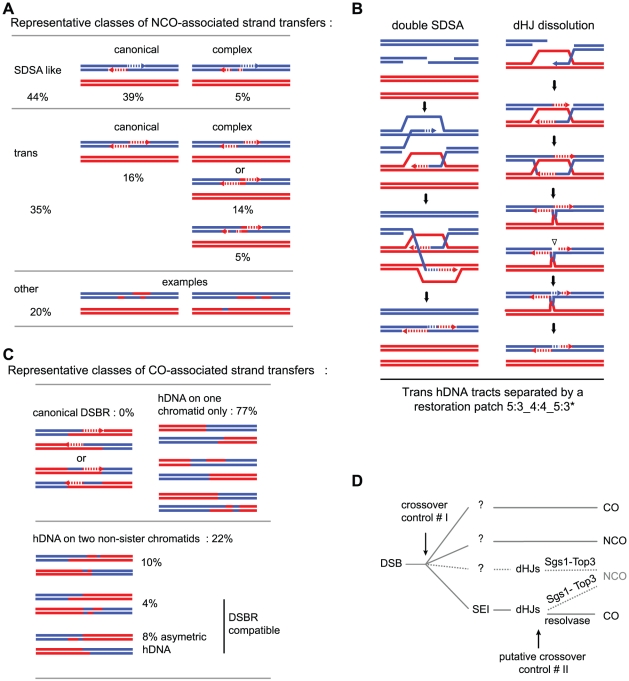
Representative classes of NCO- and CO-associated strand transfers and model for meiotic crossover control points. (A) Representative classes of NCO-associated strand transfers are expressed as percentages of total NCOs. The “SDSA-like” and the “trans” classes are divided in two sub-classes: the canonical sub-classes with the expected patterns from the canonical models (Figure 1a, b and c respectively) and the complex sub-classes with patterns presenting the expected profiles plus additional unpredicted 6∶2 or 4∶4 tracts. The “other” class contains NCOs with strand transfer patterns that cannot be attributed unambiguously to a specific origin. (B) Models for the formation of 4∶4 tracts within trans hDNAs associated to NCOs. During double SDSA, one end invades a non-sister chromatid as expected while the second end first invades the sister chromatid then a non-sister chromatid. Annealing of the two ends leads to the formation of a trans hDNA pattern with a 4∶4 tract in the middle. During dHJ dissolution, an unrepaired nick formed before or during the topological processing of the junction can induce nick translation, which generates a 4∶4 tract. (C) Representative classes of CO-associated strand transfers are presented as percentages of total COs with detectable strand transfer. For the “canonical DSBR” class, the two expected patterns are represented (see Figure 1e). The “hDNA on one chromatid” class and the three sub-classes of “hDNA on two non-sister chromatids” are illustrated by examples of observed patterns. The two “DSBR compatible” sub-classes of hDNA on two non-sister chromatids correspond to situations where the strand transfers from the two non-sister chromatids do not overlap. (D) In addition to the first CO control point before or during the transition between DSB and invasion of the homolog by one end of the DSB, our observations support a model where dHJs can be dissolved into NCOs and therefore constitute a second CO control point. The question marks indicate that except SEIs, no recombination intermediates have been isolated for the other pathways.

Based on the patterns of the persistent marks of strand transfer events, we identified 169 NCOs in two meioses in the absence of Msh2 (Figure 4A). Out of these, 75 (44%) present a strand transfer pattern compatible with the simple SDSA repair pathway (Table 1), 59 (35%) present a pattern compatible with dHJ dissolution or double SDSA repair pathways (Table 2) and the remaining 35 (21%) present patterns impossible to attribute unambiguously to a particular origin (Table 3). These results confirm that the simple SDSA pathway is a major contributor to meiotic NCOs. In addition to this pathway, one unprecedented feature is the quantitative abundance of trans hDNA associated with NCOs, which are almost as frequent. Other studies from the *S. cerevisiae ARG4* locus using poorly repairable hairpin extruding palindromes [25] and from a Drosophila m*sh6* mutant [65] also reported a significant level of trans hDNA associated with NCOs, suggesting a conserved mechanism. Interestingly, studies carried out at the *HIS4* locus using either poorly repairable hairpins [37] or MMR deficient mutants [55] also revealed trans hDNAs but at a much lower frequency compared to us, and almost half of those events were associated with COs, which is not what we observed. Combined with ours, these results suggest that the frequency and the nature of the trans hDNAs may vary according to the locus.

**Table 1:** NCO-associated *msh2*Δ strand transfers: SDSA-like strand transfer patterns.

NCO-associated SDSA-like strand transfer pattern	occurrence
3∶5	66
3∶5_4∶4_3:5	5
5∶3_6∶2_5∶3	1
3∶5_4:4_3∶5_4∶4_3∶5	1
5∶3_4∶4_5∶3_4∶4_5∶3	1
3∶5_2∶6_3∶5_2∶6_3∶5	1
total	75

We included in the hDNA patterns the distribution profile of the eight DNA strands over the entire length of the NCO events. Underscores indicate alternations in the distribution profile of the DNA strands in a given event.

**Table 2:** NCO-associated *msh2*Δ strand transfers: trans hDNA-like strand transfer patterns.

NCO-associated trans hDNA-like strand transfer pattern	occurrence
3∶5_3∶5* a	28
3∶5_4∶4_3∶5*	22
3∶5_2∶6_3∶5*	2
3∶5_4∶4_3∶5_3∶5*	1
5∶3_4∶4_5∶3_5∶3*	1
3∶5_3∶5*_4∶4_3∶5*	1
5∶3_4∶4_5∶3*_4∶4_5∶3*	1
5∶3_4∶4_5∶3_4∶4_5∶3*	1
3∶5_4∶4_3∶5_4∶4_3∶5_3∶5*	1
5∶3_4∶4_6∶2_5∶3*_6∶2_5∶3*	1
Total	59

a An asterisk accompanying a 3∶5 or 5∶3 tract differentiates within a single pattern two tracts with the same global strand asymmetry but with different strand distributions. Trans hDNAs are defined by two such hDNA tracts on the same chromatid.

**Table 3:** NCO-associated *msh2*Δ strand transfers: other strand transfer patterns.

NCO-associated other strand transfer pattern	occurence	single chromatid	two non sisters	two sisters
3∶5	1		1	
2∶6	8	8		
3∶5_2∶6	5	5		
4∶4* b _3∶5	1		1	
2∶6_1∶7	1			1
3∶5_4∶4_2∶6	4	3		1
6∶2_4∶4_6∶2	1	1		
3∶5_4∶4_4∶4*	1		1	
5∶3_4∶4_3∶5	2		2	
3∶5_4∶4_3∶5*	1			1
4∶4*_3∶5_4∶4*	1		1	
3∶5_2∶6_4∶4_3∶5	1	1		
2∶6_3∶5_2∶6_3∶5*	1	1		
6∶2_5∶3_4∶4_3∶5	1		1	
5∶3_4∶4_5∶3*_6∶2_5:3*	1	1		
3∶5_4∶4_2∶6_3∶5_2∶6	1	1		
3∶5_4∶4_3∶5_2∶6_3∶5	1	1		
5∶3_4∶4_3∶5_4∶4_3∶5	1		1	
4∶4*_5∶3_6∶2_5∶3*_6:2_5∶3*	1		1	
3∶5_2∶6_3∶5_2∶6_3∶5_2∶6_3∶5_2∶6_3∶5_2∶6	1	1		
total	35	23	9	3

b 4∶4* tracts are aberrant 4∶4 tracts with symmetric hDNAs.

### A minor fraction of SDSA events are complex

Among the 75 NCOs compatible with the simple SDSA repair pathway (Table 1 and Figure 4A), 66 exhibit a continuous 5∶3 hDNA pattern on one strand only exactly as predicted by the canonical SDSA pathway. The other 9 show a discontinuous 5∶3 hDNA pattern, interrupted by 4∶4 (7 cases) or 6∶2 (2 cases) tracts. Both 4∶4 and 6∶2 tracts can result from Msh2-independent mismatch repair toward restoration and full conversion respectively as already described [36], [55], [66], [67]. One possible mechanism for 4∶4 tract generation in the absence of Msh2 consists in two successive template switches during SDSA. The first switch would go from the non-sister chromatid to the sister or the parental chromatid, and the second switch from the sister or the parental chromatid back to the non-sister chromatid. Template switches have already been observed both in meiotic [68] and mitotic cells [69] at comparable frequencies (about 10%).

### Msh2-independent repair patches within trans hDNAs suggest that dHJ dissolution contributes significantly to meiotic NCO formation

Out of the 59 NCOs compatible with dHJ dissolution or double SDSA repair pathway (Table 2 and Figure 4A), 28 exhibit a continuous trans hDNA pattern on the same chromatid that we called 5∶3_5∶3* to indicate that the two consecutive 5∶3 tracts are different. Interestingly, 24 other NCOs exhibit a trans hDNA pattern with the two opposite hDNA tracts separated by a single 4∶4 (22 cases) or 6∶2 tract (2 cases), showing a strong excess of 4∶4 tracts. Previous work from the Sekelsky laboratory already reported repair patches in between trans hDNAs but their analysis was restricted to one meiotic chromatid only [65], [70]. Finally, 7 NCOs with trans hDNA present more complex profiles similar to the complex profiles observed for simple SDSA events.

Formally, the trans hDNA pattern could result from double SDSA involving homolog invasion from the two ends of the break (Figure 1). The significant fraction of events containing a restoration patch separating the two hDNA tracts could be explained if one end first invades the sister chromatid and then undergoes a template switch to the homolog (Figure 4B). However, the template switching frequency observed in mitotic and meiotic cells is much lower than the fraction of restoration tracts observed in this trans hDNA category (about 10% vs 50%) [68], [69]. In addition, recent findings support a model in which only one end searches and invades one homologous non-sister chromatid while the other is kept with the sister [71], [72], which would disfavor the double SDSA pathway.

Alternatively, the fact that roughly half of the trans hDNAs contain a restoration patch separating the two hDNA tracts could indicate the occurrence of a recombination intermediate containing an entry point for Msh2-independent mismatch repair specifically located close to the junction of the hDNA tracts. Such an entry point could be a nick abnormally left unrepaired after the combined action of a helicase and a topoisomerase I during the dissolution of a dHJ. Such a nick could be used as a primer for DNA synthesis leading to Msh2-independent mismatch repair (Figure 1 and Figure 4B). Interestingly, nicks that would have resulted from double SDSA are not expected in between but on both sides of the hDNA tracts and their repair would have induced a different pattern. Radford et al. identified trans hDNAs in the *D. melanogaster mei-9* mutant. Because *MEI-9* is essential to CO formation and it encodes an ortholog of the *S. cerevisiae* Rad1 endonuclease, the authors proposed that unresolved dHJs may lead to NCOs by dissolution [70].

In conclusion, our observations suggest that at least part of trans hDNAs come from dissolution of dHJs. dHJs would therefore constitute a novel putative control point for CO versus NCO formation (Figure 4D).

### How to reconcile NCO formation through Sgs1-Top3 dHJ dissolution with current meiotic recombination models?

The hypothesis that a significant fraction of dHJs give rise to NCOs appears inconsistent with the fact that NCOs level is not affected either by the absence of the transcription factor Ndt80 that induces the accumulation of unresolved joint molecules (JMs) or by the absence of the ZMM proteins that strongly impedes JMs formation [7], [9], [73]. These observations led to the model that dHJs do not give rise to NCOs but only to COs. We envision two possibilities to reconcile this hypothesis to ours. The first possibility is to suggest that only the fraction of JMs that is meant to become COs by dHJ resolution did not form or remained unresolved and accumulated in the absence of the ZMM proteins or Ndt80, respectively. A minor fraction of JMs engaged to dHJ dissolution would still form and be processed properly as NCOs. The second possibility is that another type of recombination intermediates that has not been detected so far yields dHJs that are meant to become NCOs by dHJ dissolution (Figure 4D). The hypothesis that a significant fraction of dHJs could give rise to NCOs is supported by the increase in COs in the absence of Sgs1, known to catalyze dHJ dissolution *in vitro* in combination with the type I topoisomerase Top3 [45]. This increase in COs has been observed in mitosis [74] and in meiosis both at specific loci [75] and genome-wide [45]. Further support comes from the observation that Sgs1 deletion in the ZMM mutants rescues their CO defect. This led to a model in which the ZMM proteins would stabilize and protect early recombination intermediates from the action of Sgs1 [73], [76], [77]. Moreover it has been observed that TOP3alpha/Top3 and BLAP75/Rmi1, which act together with RECQ4A/Sgs1 to unwind a dHJ *in vitro* [26], are essential for proper meiotic progression in *A. thaliana* [78]-[80].

### Only a minor fraction of NCOs could result from dHJ resolution

Out of the 35 remaining NCOs (Table 3 and Figure S3), 13 present relatively simple strand transfer patterns composed of a 6∶2 tract, associated with a 5∶3 tract in 5 cases. These events have been considered separately from the two previous classes because each of them could have arisen from either pathway, with 6∶2 tracts resulting from either double-strand gap repair or full conversion. 9 out of the 35 present hDNA tracts on two homologous chromatids, with 4 of them having two overlapping hDNA tracts reminiscent of Holliday junction branch migration within a homoduplex DNA that forms symmetric hDNAs (aberrant 4∶4* tracts) (Table 3). This confirms that the dHJ pathway poorly contributes to NCO formation as was previously observed [37]. The 13 remaining NCOs present more complex strand transfer patterns coming from multiple putative origins.

### Asymmetric positioning of recombination intermediates around DSBs and/or D-loop and dHJ migration can lead to asymmetric hDNA tracts at CO sites

Out of 181 COs from the two *msh2Δ* meioses for which hDNAs have been analyzed, 155 were associated with strand transfers (Table 4, Figure 4C, and Figure S4). Surprisingly, all the strand transfer patterns were different from the pattern predicted by the canonical CO pathways, i.e. two continuous 5∶3 tracts distributed on the two non-sister chromatids around the DSB site (Figure 1). Only 36 COs out of 155 (23%) carried hDNA on the two non-sister recombining chromatids, and only 19 (12%) of them presented a strand transfer pattern compatible with the outcome of the canonical CO pathways (Table 5). Remarkably, 13 out of these 19 COs presented clearly asymmetric hDNA tract length, with a long hDNA tract on one chromatid and a short hDNA tract on the other chromatid (Table 5, and see example Figure S2C). This hDNA tract asymmetry is consistent with previous genetic studies that reported infrequent co-conversion of markers flanking a single DSB hot spot [81]. Such an asymmetry could reflect an asymmetric positioning of recombination intermediates around DSBs resulting from either a limited strand invasion combined to an extensive DNA synthesis, or an extensive strand invasion combined to a limited DNA synthesis, as proposed by Jessop et al. [81].

**Table 4:** CO-associated *msh2Δ* strand transfer patterns.

CO-associated strand transfer pattern	occurrence	single chromatid	two non-sister chromatids
none	26		
2∶6	26	26	
3∶5	16	16	
4∶4*	1		1
3∶5_2∶6	15	15	
3∶5_4∶4	7	7	
2∶6_4∶4	5	5	
3∶5_4∶4*	4		4
5∶3_5∶3*	1	1	
3∶5_5∶3	2	2	
5∶3_2∶6	1	1	
3∶5_4∶4_3∶5	2	2	
2∶6_4∶4_2∶6	4	4	
3∶5_4∶4_2∶6	5	5	
2∶6_3∶5_4∶4	2	2	
3∶5_2∶6_3∶5*	3		3
3∶5_4∶4_3∶5*	2		2
2∶6_4∶4*_3∶5	1		1
4∶4*_3∶5_3∶5*	1		1
5∶3_5∶3*_6∶2	2		2
3∶5_4∶4_5∶3	2	2	
3∶5_6∶2_5∶3	1	1	
2∶6_3∶5_5∶3	1	1	
6∶2_4∶4_3∶5	2	2	
> 3 DNA tracts	49	27	22
total	181	119	36

**Table 5:** Comparison of some properties of CO- and NCO-associated strand transfer patterns.

	CO	NCO
class 4∶4	72	51
class 4∶4*	12	4
class 6∶2	104	31
class 3∶5_5∶3	26	4
hDNA on one chromatid only	119	157
hDNA on both homologous chromatids	35	9
DSBR like (all)	19	0
DSBR like with asymmetric hDNAs	13	0

**Figure 5:**
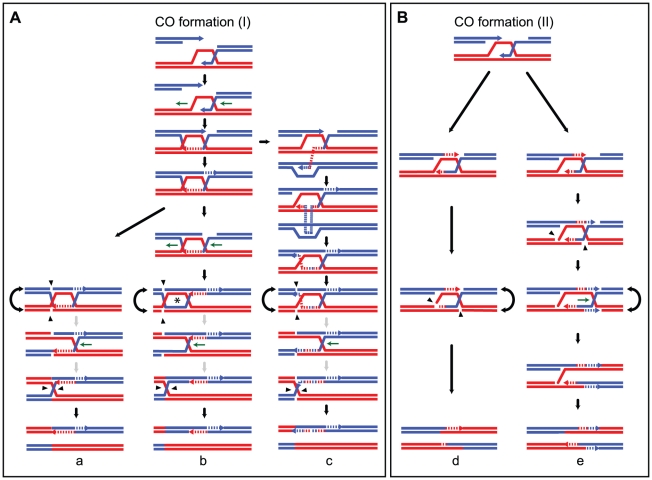
Models for meiotic CO formation. CO formation (I) and CO formation (II) are variations of the canonical models that include ligated and non ligated dHJs respectively. (A) Model for strand transfers on only one chromatid after D-loop and dHJ migration. DNA synthesis primed at the first invading end is coupled with migration of the D-loop in the same way (horizontal green arrows). Migration of the D-loop can partially (not shown) or completely erase the first hDNA formed after strand invasion as shown here. The ligated dHJ can be resolved (a) or can keep migrating away from the invasion point (b and c). dHJ migration within a homoduplex DNA generates an aberrant 4∶4 tract or symmetric hDNA tract (indicated by a star), that corresponds to the region encompassed by the two HJs. If dHJ migration is long enough, it can also create a 4∶4 tract located in between the hDNA formed at the initial Spo11-DSB and the closest HJ. Remarkably, aberrant 4∶4 tracts are almost never detected, suggesting they are very small. This implies that the two HJs are cleaved when they are close to one another, in a one- or two-step process as illustrated here. The grey arrows indicate that the two-step resolution process is speculative and as equally probable as a one-step resolution process of two close HJs. (c) illustrates template switching between non-sister and sister chromatids that generates an alternation in strand transfer on a single chromatid. Importantly, migration of the D-loop and dHJ could also occur in the opposite direction compared to the one presented here, but it would lead to the same outcome and has not been represented for simplicity. (B) Models for asymmetric and complex strand transfers on two non-sister chromatids. Asymmetric positioning of recombination intermediates around the initial Spo11-DSB can generate one long and one short hDNA tracts. The asymmetry can come from either a long DNA end invasion and a short DNA synthesis (d) or a short DNA end invasion and a long DNA synthesis (not shown) [81]. Note that such asymmetry can also affect CO formation as proposed in panel A. After first end invasion and second end capture, panel (d) shows the cleavage of the recombination intermediate by a nuclease such as the structure-specific endonuclease Mus81 to generate a CO. Panel (e), depicts how DNA synthesis initiated at transient nicks present in recombination intermediates combined with HJ migration can lead to the inversion of a 5∶3 hDNA tract into a 3∶5 hDNA causing strand transfers associated with COs to be more complex. DNA is cut close to the HJ resulting from the first end invasion by a nuclease such as the structure-specific endonuclease Mus81. DNA synthesis is subsequently initiated at that nick. The invading strand that has already been used as a template for elongation of the second DSB end is also used as a template from that nick thanks to HJ branch migration. Under this scenario, a 5∶3 segregation tract can be converted into an opposite 3∶5 tract. Note that template switching as proposed in (A, c) may also increase strand transfer complexity.

Alternatively, hDNA tract length asymmetry could result from migration of the D-loop [82] after the first strand invasion and extension. D-loop migration could partially or completely dismantle the first hDNA formed (Figure 5A). Unexpectedly, we observed that the majority of strand transfer tracts of COs (119/155 ie 77%) are present on one chromatid only (Figure 4C, Figure S2A and S2B, Figure S4, Table 4, and Table 5). In many cases these events occurred in regions with high marker density, ruling out detection artifacts due to local lack of markers to explain this asymmetry. The migrating D-loop model could also explain this asymmetric distribution of strand transfer tracts onto the recombining chromatids at CO sites (Figure 5).

Formation of a stable single end invasion (SEI) intermediate prior to CO formation [10], [33], [81] could favor D-loop migration. More specifically, it is likely that the first invading end is extended prior to capture of the second end, although direct evidence is still lacking. This would leave the opportunity for the corresponding junction to migrate in either direction with respect to the invasion point and therefore affect the size of the corresponding hDNA up to its disappearance (Figure 5).

In situations where D-loop migration is followed by dHJ formation and migration, 4∶4 tracts and aberrant 4∶4 tracts are expected to form. This scenario is supported by the frequent strand transfer patterns composed of a single 5∶3 or 6∶2 tract associated with a 4∶4 tract (Table 4 and Figure S4), which cannot simply result from an asymmetric positioning of recombination intermediates around the DSB. Interestingly, these observations are also consistent with what can be observed in wild type meioses. As shown in Table 6, nine COs are separated from their associated 3∶1 gene conversion by a 2∶2 segregating tract. Savage and Hastings also observed such a pattern by performing a systematic analysis of the segregation patterns of multiple markers at the *S. cerevisiae HIS1* locus [83]. Nevertheless, aberrant 4∶4 tracts are infrequent. One possible explanation for this puzzling observation is that the source of aberrant 4∶4 tracts, which is the region between the two HJs, is so small after dHJ migration that it is hardly detectable (Figure 5A). This explanation is compatible with the inter-junction distance measured for dHJs visualized by electron microscopy that averages 260 bp [18], [84]. One can also imagine that aberrant 4∶4 tracts within dHJs disappear by branch migration during a putative sequential resolution of the two HJs (Figure 5A). Finally, we cannot formally exclude Msh2-independent mismatch repair as a source of asymmetry in strand transfer distribution at CO sites.

**Table 6:** CO-associated strand transfers in the presence of Msh2.

CO-associated strand transfers	occurrence
none	62
3∶1	194
3∶1_2∶2	8
3∶1_1∶3	4
3∶1_2∶2_3∶1	11
3∶1_2∶2_3∶1*_2∶2	1
3∶1_2∶2_1∶3_2∶2*_1∶3	1
3∶1_2∶2_3∶1_2∶2_3∶1_2∶2_3∶1	1
total	282

Very interestingly, we did not observe asymmetry in strand transfer tract length at NCOs with trans hDNAs (data not shown), for which we can assume rather confidently that the initiating DSBs are located at the boundary between the hDNA patches. These apparently contradictory observations raise an interesting question: are dHJs dissolved into NCOs different from dHJs resolved into COs? Multiple observations could support such a difference. First, Pan et al. [62] recently proposed that at least part of DSB ends may be processed asymmetrically. Formation and maturation of the dHJ could depend on the nature of the invading end i.e. with long or short single-stranded tail. Alternatively, symmetric or asymmetric processing of the two ends of a given DSB could generate different types of dHJs. Second, it has been recently proposed that the two ends of a DSB were sequentially released to interact with the homolog [71]. Formation and maturation of the dHJ could depend on the release of the second end. Third, maturation of the dHJ could depend on the properties of the D-loop. D-loop migration could be necessary to form stable CO intermediates (SEI). Finally, it is possible that formation and maturation of dHJs depend on the combined action of all these factors.

### COs are associated with complex strand transfers in the absence of MMR

Previous work pointed out the complexity of CO-associated strand transfer patterns with a limited number of markers [37]. Taking advantage of a greater marker density, we confirmed such a complexity and revealed patterns even more complex. Among 155 strand transfer patterns associated with COs, 112 comprised between 2 to 8 successive DNA tracts including 49 patterns comprising more than 3 successive DNA tracts (Table 4). This complexity results from the accumulation of 6∶2 and 4∶4 tracts in between 5∶3 tracts. As seen above, NCO-associated hDNA patterns also show marks of Msh2-independent mismatch repair but more than half of them are as expected without additional marks of complex events. This shows that CO intermediates present specificities that make them prone to complex events, notably through Msh2-independent mismatch repair [36], [55], [66], [67].

As already mentioned, branch migration of HJs is a source of 4∶4 tracts associated with COs, as well as template switches between non-sister and sister chromatids. The repair of gaps is a source of 6∶2 tracts. In that respect, it is interesting to note that 6∶2 tracts are more frequently associated with COs (104; 67%) compared to NCOs (31; 19%) (Table 5). It suggests that gaps between DSB ends could preferentially be repaired by a CO pathway. Such gaps may arise from two very close Spo11-induced DSBs or from 3′ end removal most likely after invasion of a homologous sequence.

As for dHJ dissolution, we propose that entry points for Msh2-independent mismatch repair could also be nicks in dHJ intermediates. Such nicks could lead to either 6∶2 or 4∶4 patches if they are used to prime DNA synthesis, as well as the more complex 5∶3_3∶5 pattern (see below). These nicks could be intrinsic properties of dHJs that could exist under a non-ligated form [85]. They could also result from the action of structure-specific nucleases during, or independently, of the resolution process. Alternatively, such nicks could also result from Msh2-independent processing of mismatches (Figure 5).

In conclusion, although compatible with dHJ-containing recombination intermediates [86], the majority of CO-associated hDNA patterns are made more complex by frequent Msh2-independent mismatch repair [36], [55], [66], [67] that could result from a combination of factors including repair of double stranded gaps, repair of nicks, template switches between non-sister and sister chromatids, and HJ branch migration.

### A model for generating opposite hDNA tracts at a single DSB after nick translation and HJ branch migration

The most puzzling CO-associated hDNA pattern that we observed comprises opposite hDNA tracts i.e. 5∶3_3∶5 tracts in 26 cases (17%) while it is almost never associated with NCOs (4 cases, 2%) (Table 5). Such a pattern is usually interpreted as resulting from two recombination events at the same locus. We reasoned that under this scenario at least three chromatids should frequently be involved. However, this was never the case, suggesting that the 5∶3_3∶5 pattern results mainly from the repair of only one DSB. We propose that nick translation combined with HJ branch migration can transform a 5∶3 tract into the opposite 3∶5 configuration (Figure 5Be).

### Shorter strand transfers in the absence of MMR suggest that transient recombination intermediates are repaired in the presence of MMR

In the presence of MMR, hDNAs are repaired and can lead to full conversions. In the absence of MMR, only hDNAs associated with the final recombination products can be revealed but not the transient ones. Comparing the lengths of the strand transfers in the presence and absence of Msh2 can therefore be informative about the processing of hDNAs. The strand transfer tract size considered for a given CO or NCO in the absence of Msh2 corresponds to the sum of the length of all the individual 5∶3, 6∶2 and 4∶4 patches associated with the event.

**Figure 6:**
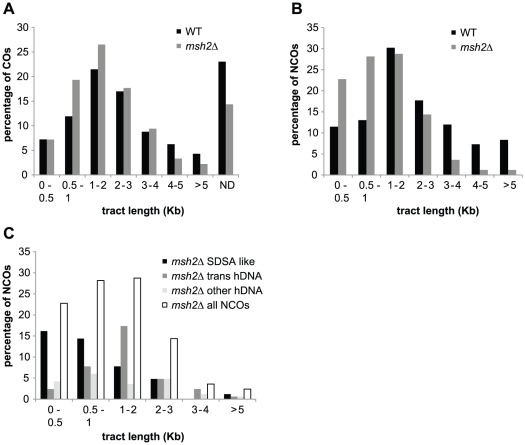
hDNA and gene conversion tract length. (A) Percentage of strand transfers associated with COs from wild type (black) and *msh2Δ* meioses (grey) as a function of tract length. ND corresponds to COs without detectable DNA strand transfer. (B) Percentage of strand transfers associated with NCOs from wild type (black) and *msh2Δ* meioses (grey) as a function of tract length. (C) Percentage of strand transfers associated with the three classes of *msh2Δ* NCOs as a function of tract length. Strand transfer patterns compatible with SDSA (black); trans hDNA patterns (dark grey); all other strand transfer patterns (light grey); all strand transfer patterns combined (white).

**Figure 7:**
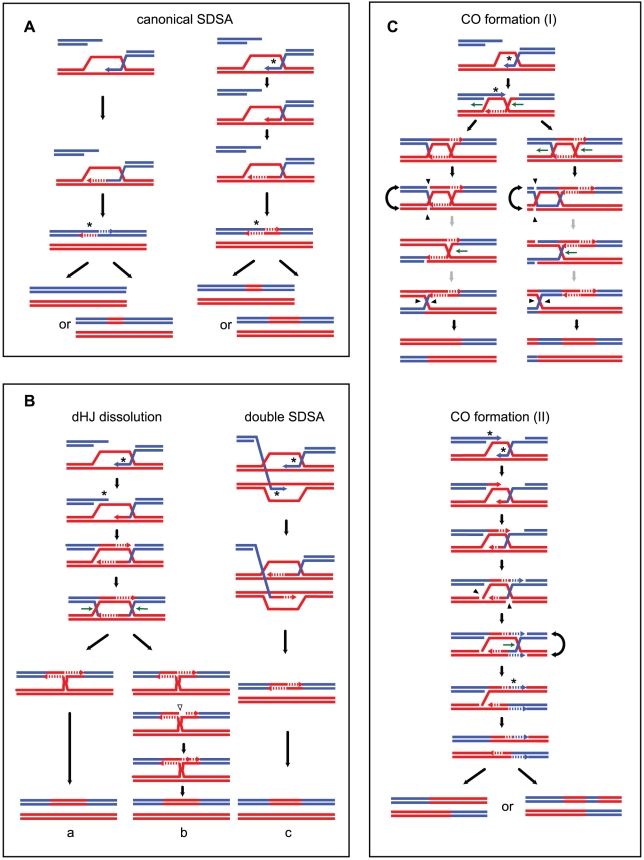
MMR–dependent conversion of transient hDNAs reconciles strand transfer patterns observed in the presence of Msh2 with those observed in the absence of Msh2. The asterisks mark the hDNAs repaired by MMR. (A) Conversion of transient hDNA during canonical SDSA. On the left, the transient hDNA formed during the first 3′ end invasion is not repaired by MMR. Conversion of the second 3′ end only produces a short patch of conversion, while its restoration produces a silent event. On the right, the invading 3′ end is converted during strand invasion. Depending on the conversion or restoration of the second 3′ end, a long or a short patch of conversion is formed. In both cases the event is detectable. (B) During dHJ dissolution and double SDSA, conversion of the invading 3′ end combined with conversion of the second 3′ end after its capture can form a uniform conversion tract as observed for most of the NCOs in the presence of MMR. In this context, repair of a nick formed before or during resolution of the junction leads to a non-detectable event (b). (C) Conversion of transient hDNA during CO formation pathways I and II. Conversion of the invading 3′ end combined with conversion of the second 3′ end after its capture can form either a uniform 3∶1 conversion tract associated or not with a 2∶2 segregation tract (pathway (I)) or uniform 3∶1 conversion tract associated or not with a 1∶3 segregation tract (pathway (II)).

We observed that the median sizes of strand transfers associated with both COs and NCOs are significantly smaller in the absence of Msh2 (Figure 6A and 6B). In the case of COs, this size difference is mild (1.6 kb vs 1.8 kb, p = 0.029 Wilcoxon test), but it is much greater for NCOs (1 kb vs 1.8kb, p = 7.7×10^−10^ Wilcoxon test). Much of the increase in total number of NCOs in the absence of Msh2 comes from a greater number of short tract events (Figure 6B). A possible explanation comes from the fact that transient hDNAs formed during recombination cannot be detected in the absence of MMR whereas they can formally be repaired and converted by MMR. As a consequence, conversion tracts are expected to be shorter in the absence of MMR (Figure 7). Interestingly, the SDSA-compatible NCOs are the major contributors to small size events in the absence of Msh2 with a median size about 2 fold smaller than the strand transfers associated with NCOs in the presence of MMR (Figure 6B and 6C). This result is compatible with frequent conversions of both hDNAs formed by strand invasion and second end capture during SDSA when Msh2 is present while the hDNA formed by strand invasion is transient and not detectable in the absence of Msh2 unlike the hDNA resulting from second end capture [87] (Figure 7).

Strand transfer tract length analysis did not allow us to determine which of the double SDSA or the dHJ dissolution pathway is the main precursor of NCOs with trans hDNAs. NCOs with trans hDNA patterns show a median length similar to the median length of CO-associated hDNA tracts. This observation is compatible with the dHJ as a common precursor. However, trans hDNA could also result from double SDSA events. We therefore considered independently the strand transfers from the two DNA strands for NCOs with trans hDNAs and found that their median sizes and size distributions are not significantly different from those of strand transfers compatible with single SDSA events (data not shown).

### Strand transfer patterns observed in the absence and in the presence of Msh2 are compatible

When present, the MMR machinery repairs mismatches formed during strand transfer by excision of one of the two DNA strands and subsequent gap fill in. Depending on which strand is repaired, the corresponding markers show either a continuous Mendelian (2∶2) or non-Mendelian (3∶1) segregation profile. We analyzed the segregation profiles of markers associated to COs or NCOs out of three meioses in the presence of MMR (Table 6 and Table 7). Among 65 NCOs, 53 (82%) presented a uniform conversion tract of 3∶1, as expected from the canonical SDSA pathway (Table 7, Figure 1, and Figure 7A). In contrast, in the absence of Msh2, only 44% of strand transfers associated with NCOs were compatible with the canonical SDSA pathway, and a high fraction (35%) showed trans hDNAs that we proposed to result from double SDSA or dHJ dissolution (Table 1, Table 2, and Figure 4A). We proposed above that the 3′ single strand ends formed at DSBs are frequently converted during strand invasion and second end capture. Under this assumption, the dHJ dissolution and double SDSA pathways are expected to form long and uniform 3∶1 segregation profiles in the presence of MMR (Figure 7B). Any nick that would form during dHJ dissolution would take place within a homoduplex DNA and its repair would therefore be undetectable (Figure 7B). Such repair is also expected to affect most hDNAs resulting from template switches between non-sister and sister chromatids and make them undetectable. Altogether, these explanations are consistent with the fact that the combined fraction of SDSA-like and trans hDNA patterns observed in the absence of Msh2 (79%) is comparable with the fraction of 3∶1 pattern observed in the presence of Msh2 (82%). The remaining NCOs with complex strand transfers that affect two non-sister chromatids observed both in the presence and absence of Msh2 could result from pathways involving dHJ resolution (Figure 1).

**Table 7:** NCO-associated strand transfers in the presence of Msh2.

NCO-associated strand transfers	occurrence
3∶1	53
3∶1_1∶3	1
3∶1_2∶2*	3
3∶1_2∶2_3∶1	5
3∶1_2∶2*_3∶1	1
3∶1_2∶2_3∶1*	1
3∶1_1∶3_1∶3*_2∶2_1∶3*	1
total	65

The asterisks indicate that the corresponding 3∶1 or 2∶2 segments are different from the other fragments with the same global segregation profiles.

Interestingly, COs formed in the presence of Msh2 also showed mainly strand transfers with uniform 3∶1 marker segregation (88%) (Table 6). We tested if the two CO formation models derived from the analysis of strand transfers in the absence of Msh2 (Figure 5) were compatible with this observation. Figure 7C describes how the repair of 3′ single strand ends during strand invasion and second end capture could lead to the formation of a uniform 3∶1 segregation pattern in both CO formation pathways in the presence of MMR. As for NCOs, such an early repair of hDNA in the presence of MMR would prevent detection of nick repair and most template switches between non-sister and sister chromatids in CO intermediates. This scenario supports the higher frequency of complex events observed in the absence versus the presence of Msh2, where 43% and 5% of the strand transfers present more than two DNA tracts, respectively (Table 4 and Table 6, and Figure 7C).

Although we cannot formally exclude that the absence of Msh2 itself could constitute a source of complexity for recombination events, we do not favor this hypothesis. The fact that the COs and NCOs strand transfer patterns observed in the absence of Msh2 can explain the strand transfer patterns observed in the presence of Msh2 supports the idea that the intermediates observed in the absence of Msh2 reflect normal intermediates.

In conclusion, the genome-wide analysis of recombination intermediates performed in the absence of Msh2 reinforces the plasticity of CO formation already anticipated from hot spots-specific studies. In particular, we showed that the majority of hDNAs associated with CO are asymmetrically distributed onto the recombining chromatids, leading us to propose variations of current recombination models. Interestingly, our study also revealed that a significant fraction of NCOs do not arise from simple SDSA, raising the idea that dHJs are also involved during NCOs formation. Further studies using specific mutants such as mutants of the RecQ helicase Sgs1 will be needed to clarify the nature of such alternative pathway(s). Because meiotic recombination is well conserved through evolution, the new findings presented in this work will have a broad impact, and serve to further enlighten this complex topic.

## Materials and Methods

### Strains and media

All yeast strains used in this study are derivatives of S288C [88] and SK1 [89]. Strain genotypes are listed in Table S1. *MSH2* disruption was performed by PCR-mediated gene replacement [90] and the sequences of the oligonucleotides used are in Table S2. SK1 x S288C crosses were made on YPD plates and diploids were subcloned onto selective plates before transferring onto 2% potassium acetate sporulation plates at 30°C. Tetrads were dissected after 3–5 days. For WT5, 6 and 7, sporulation was induced in liquid medium. The hybrids made involved the following crosses∶ NHY113 and SK1708 for wild type; BLY107 and BLY114 for *msh2Δ*.

### DNA extraction, labeling, and hybridization

Wild type tetrads were dissected and only tetrads giving four viable spores were considered for genotyping by tiling array DNA hybridization (Table S3). Note that the whole colonies arising from spore germination have been genotyped, thereby neglecting potential hybridization problems due to heterozygosities resulting from post-meiotic segregation [57]. Three control hybridizations were performed for each parental strain (Table S3).

To study hDNA in *msh2Δ* hybrids, two octads were obtained from two tetrads by separating the mother from the daughter cell after the first mitotic division of four viable spores as described in Figure 2. The third meiosis from the *msh2Δ* hybrid was only considered for CO analysis, thus only four cell populations from the four spores were genotyped.

Genomic DNA was purified from 300 ml of overnight saturated YPD culture using a Qiagen genomic-tip 500/G following the Qiagen genomic DNA handbook with the slight modification of extending zymolyase and protease K digestion to 1 hour. 12 µg of genomic DNA were fragmented by DNaseI treatment, biotin-end-labeled and hybridized to Affymetrix S. cerevisiae Tiling 1.0R Array as described in [50].

### Data analysis and genotyping

We genotyped spores using ssGenotyping [46], [49]. The genotypes are provided in Table S4. SK1 sequence was obtained from the Saccharomyces Genome Resequencing Project [51]. SK1-S288C genome alignment has been performed using LAGAN [91]. To prevent the consideration of artificial polymorphisms using ssGenotyping, alignments have been modified by replacing the Ns from the SK1 sequence by the corresponding S288C sequence when it was either A, C, G or T. Annotation of every single CO and NCO has been curated manually using tetrad inspector from the ssGenotyping package. Graphical views of recombination events are available upon request. Recombination events identified by only one marker (Table S5) were discarded. Events identified by 4∶0 segregating markers were considered to be of mitotic origin and were not taken into account. Conversion tracts lengths are tract size estimates obtained using midpoints of flanking inter-marker intervals. Events separated by less than 5 kb were considered to have arisen from the same DSB and were therefore combined. This rule was not applied when a gene conversion occurred on a chromatid not involved in a CO but located less than 5 kb away from the CO. In this latter case, the gene conversion was considered separately from the CO.

To analyze *msh2Δ* octads, we arbitrarily divided each octad in two tetrads that were analyzed independently with ssGenotyping (see Figure 2). Recombination events were reconstituted manually by combining the genotypes of the two arbitrary tetrads.

Raw data are available from ArrayExpress (http://www.ebi.ac.uk/arrayexpress) under accession number E-MTAB-508.

## Supporting Information

Figure S1 Examples of NCOs-associated strand transfer events observed in the absence of Msh2. The red and blue vertical bars represent markers with the S288C and SK1 genotypes respectively along the chromosomes. The genotype of the mother and daughter cells from the first mitotic division of each of the four spores (1,2,3,4) reflects the DNA content of each chromatid from these spores (see Figure 2). The approach does not allow distinguishing which strand was the upper or the lower strand during the recombination event. (A) “trans” event associated with a NCO with a typical 5∶3_5∶3* segregation pattern. (B) “trans” event associated with a NCO with an additional 4∶4 segregation tract in between the 5∶3 and 5∶3* tracts.
(EPS)

Figure S2 Examples of COs-associated strand transfer events observed in the absence of Msh2. Same general comments as in Figure S1. (A) and (B) show CO-associated strand transfers affecting one chromatid only. (A) A 5:3 tract associated with a 4:4 tract compose the strand transfer pattern. (B) Complex strand transfer pattern composed of a succession of 4:4_5:3_4:4_6:2_5:3* segregation tracts. (C) strand transfers affecting two non-sister chromatids with a short hDNA on the left and a long hDNA on the right with a 5:3_5:3*_6:2 segregation pattern.
(EPS)

Figure S3 Illustration of NCO-associated complex strand transfers. Tracts are not drawn to scale.
(EPS)

Figure S4 Illustration of CO-associated strand transfers that contain up to three distinct segregation tracts. Tracts are not drawn to scale.
(EPS)

Table S1 List of strains.
(DOC)

Table S2 Sequences of the oligonucleotides used to disrupt *MSH2.*
(DOC)

Table S3 List of array hybridizations.
(DOC)

Table S4 Marker coordinates and genotype calls for all spores. The index column indicates the index of the marker on the corresponding chromosome. The Ali_pos column indicates the position of the marker in the alignment of the two genomes. The S288C_pos indicates the position of the marker in the S288C genome. The type column contains S for single nucleotide polymorphism, I for insertions, D for deletions. A genotype call of 1 corresponds to S288c, and 0 corresponds to SK1. Genotype call column headers give tetrad type (wt and msh2), tetrad number, and spore letter.
(TXT)

Table S5 One marker based events.
(DOC)

## References

[1] Page SL, Hawley RS (2003). Chromosome choreography: the meiotic ballet.. Science.

[2] Arora C, Kee K, Maleki S, Keeney S (2004). Antiviral protein Ski8 is a direct partner of Spo11 in meiotic DNA break formation, independent of its cytoplasmic role in RNA metabolism.. Mol Cell.

[3] Bergerat A, de Massy B, Gadelle D, Varoutas PC, Nicolas A (1997). An atypical topoisomerase II from *Archaea* with implications for meiotic recombination.. Nature.

[4] Keeney S, Giroux CN, Kleckner N (1997). Meiosis-specific DNA double-strand breaks are catalyzed by Spo11, a member of a widely conserved protein family.. Cell.

[5] Neale MJ, Pan J, Keeney S (2005). Endonucleolytic processing of covalent protein-linked DNA double-strand breaks.. Nature.

[6] Sun H, Treco D, Szostak JW (1991). Extensive 3′-overhanging, single-stranded DNA associated with the meiosis-specific double-strand breaks at the *ARG4* recombination initiation site.. Cell.

[7] Allers T, Lichten M (2001). Differential timing and control of noncrossover and crossover recombination during meiosis.. Cell.

[8] Bishop DK, Zickler D (2004). Early decision; meiotic crossover interference prior to stable strand exchange and synapsis.. Cell.

[9] Borner GV, Kleckner N, Hunter N (2004). Crossover/noncrossover differentiation, synaptonemal complex formation, and regulatory surveillance at the leptotene/zygotene transition of meiosis.. Cell.

[10] Hunter N, Kleckner N (2001). The single-end invasion: an asymmetric intermediate at the double-strand break to double-Holliday junction transition of meiotic recombination.. Cell.

[11] Argueso JL, Wanat J, Gemici Z, Alani E (2004). Competing crossover pathways act during meiosis in *Saccharomyces cerevisiae*.. Genetics.

[12] de los Santos T, Hunter N, Lee C, Larkin B, Loidl J (2003). The Mus81/Mms4 endonuclease acts independently of double-Holliday junction resolution to promote a distinct subset of crossovers during meiosis in budding yeast.. Genetics.

[13] Lynn A, Soucek R, Borner GV (2007). ZMM proteins during meiosis: crossover artists at work.. Chromosome Res.

[14] Colaiacovo MP, MacQueen AJ, Martinez-Perez E, McDonald K, Adamo A (2003). Synaptonemal complex assembly in *C. elegans* is dispensable for loading strand-exchange proteins but critical for proper completion of recombination.. Dev Cell.

[15] Zickler D, Kleckner N (1999). Meiotic chromosomes: integrating structure and function.. Annu Rev Genet.

[16] Gaillard PH, Noguchi E, Shanahan P, Russell P (2003). The endogenous Mus81-Eme1 complex resolves Holliday junctions by a nick and counternick mechanism.. Mol Cell.

[17] Osman F, Dixon J, Doe CL, Whitby MC (2003). Generating crossovers by resolution of nicked Holliday junctions: a role for Mus81-Eme1 in meiosis.. Mol Cell.

[18] Cromie GA, Hyppa RW, Taylor AF, Zakharyevich K, Hunter N (2006). Single Holliday junctions are intermediates of meiotic recombination.. Cell.

[19] Martinez-Perez E, Colaiacovo MP (2009). Distribution of meiotic recombination events: talking to your neighbors.. Curr Opin Genet Dev.

[20] Martini E, Diaz RL, Hunter N, Keeney S (2006). Crossover homeostasis in yeast meiosis.. Cell.

[21] Szostak JW, Orr-Weaver TL, Rothstein RJ, Stahl FW (1983). The double-strand-break repair model for recombination.. Cell.

[22] Nassif N, Penney J, Pal S, Engels WR, Gloor GB (1994). Efficient copying of nonhomologous sequences from ectopic sites via P-element-induced gap repair.. Mol Cell Biol.

[23] McMahill MS, Sham CW, Bishop DK (2007). Synthesis-dependent strand annealing in meiosis.. PLoS Biol.

[24] Paques F, Haber JE (1999). Multiple pathways of recombination induced by double-strand breaks in *Saccharomyces cerevisiae*.. Microbiology & Molecular Biology Reviews.

[25] Gilbertson LA, Stahl FW (1996). A test of the double-strand break repair model for meiotic recombination in *Saccharomyces cerevisiae*.. Genetics.

[26] Cejka P, Plank JL, Bachrati CZ, Hickson ID, Kowalczykowski SC (2010). Rmi1 stimulates decatenation of double Holliday junctions during dissolution by Sgs1-Top3..

[27] Wu L, Hickson ID (2003). The Bloom's syndrome helicase suppresses crossing over during homologous recombination.. Nature.

[28] Holliday R (1964). A mechanism for gene conversion in fungi.. Genet Res.

[29] Meselson MS, Radding CM (1975). A general model for genetic recombination.. Proc Natl Acad Sci U S A.

[30] Bishop DK, Williamson MS, Fogel S, Kolodner RD (1987). The role of heteroduplex correction in gene conversion in *Saccharomyces cerevisiae*.. Nature.

[31] White JH, Lusnak K, Fogel S (1985). Mismatch-specific post-meiotic segregation frequency in yeast suggests a heteroduplex recombination intermediate.. Nature.

[32] Williamson MS, Game JC, Fogel S (1985). Meiotic gene conversion mutants in *Saccharomyces cerevisiae*. I. Isolation and characterization of pms1-1 and pms1-2.. Genetics.

[33] Allers T, Lichten M (2001). Intermediates of yeast meiotic recombination contain heteroduplex DNA.. Mol Cell.

[34] Evans E, Alani E (2000). Roles for mismatch repair factors in regulating genetic recombination.. Mol Cell Biol.

[35] Haber JE, Lankenau READ-H (2007). Evolution of models of homologous recombination.. Genome Dynamics and Stability.

[36] Hoffmann ER, Eriksson E, Herbert BJ, Borts RH (2005). MLH1 and MSH2 promote the symmetry of double-strand break repair events at the *HIS4* hotspot in *Saccharomyces cerevisiae*.. Genetics.

[37] Merker JD, Dominska M, Petes TD (2003). Patterns of heteroduplex formation associated with the initiation of meiotic recombination in the yeast *Saccharomyces cerevisiae*.. Genetics.

[38] Claverys JP, Lacks SA (1986). Heteroduplex deoxyribonucleic acid base mismatch repair in bacteria.. Microbiol Rev.

[39] Mejean V, Claverys JP (1984). Effect of mismatched base pairs on the fate of donor DNA in transformation of *Streptococcus pneumoniae*.. Mol Gen Genet.

[40] Matic I, Radman M, Rayssiguier C (1994). Structure of recombinants from conjugational crosses between *Escherichia coli* donor and mismatch-repair deficient *Salmonella typhimurium* recipients.. Genetics.

[41] Rayssiguier C, Thaler DS, Radman M (1989). The barrier to recombination between *Escherichia coli* and *Salmonella typhimurium* is disrupted in mismatch-repair mutants.. Nature.

[42] Vulic M, Dionisio F, Taddei F, Radman M (1997). Molecular keys to speciation: DNA polymorphism and the control of genetic exchange in enterobacteria.. Proc Natl Acad Sci U S A.

[43] Datta A, Hendrix M, Lipsitch M, Jinks-Robertson S (1997). Dual roles for DNA sequence identity and the mismatch repair system in the regulation of mitotic crossing-over in yeast.. Proc Natl Acad Sci U S A.

[44] Chen W, Jinks-Robertson S (1999). The role of the mismatch repair machinery in regulating mitotic and meiotic recombination between diverged sequences in yeast.. Genetics.

[45] Chen SY, Tsubouchi T, Rockmill B, Sandler JS, Richards DR (2008). Global analysis of the meiotic crossover landscape.. Dev Cell.

[46] Mancera E, Bourgon R, Brozzi A, Huber W, Steinmetz LM (2008). High-resolution mapping of meiotic crossovers and non-crossovers in yeast.. Nature.

[47] Qi J, Wijeratne AJ, Tomsho LP, Hu Y, Schuster SC (2009). Characterization of meiotic crossovers and gene conversion by whole-genome sequencing in *Saccharomyces cerevisiae*.. BMC Genomics.

[48] Winzeler EA, Richards DR, Conway AR, Goldstein AL, Kalman S (1998). Direct allelic variation scanning of the yeast genome.. Science.

[49] Bourgon R, Mancera E, Brozzi A, Steinmetz LM, Huber W (2009). Array-based genotyping in *S. cerevisiae* using semi-supervised clustering.. Bioinformatics.

[50] Gresham D, Ruderfer DM, Pratt SC, Schacherer J, Dunham MJ (2006). Genome-wide detection of polymorphisms at nucleotide resolution with a single DNA microarray.. Science.

[51] Liti G, Carter DM, Moses AM, Warringer J, Parts L (2009). Population genomics of domestic and wild yeasts.. Nature.

[52] Nishant KT, Wei W, Mancera E, Argueso JL, Schlattl A (2010). The baker's yeast diploid genome is remarkably stable in vegetative growth and meiosis.. PLoS Genet.

[53] Wei W, McCusker JH, Hyman RW, Jones T, Ning Y (2007). Genome sequencing and comparative analysis of *Saccharomyces cerevisiae* strain YJM789.. Proc Natl Acad Sci U S A.

[54] Schacherer J, Shapiro JA, Ruderfer DM, Kruglyak L (2009). Comprehensive polymorphism survey elucidates population structure of *Saccharomyces cerevisiae*.. Nature.

[55] Hoffmann ER, Borts RH (2005). Trans events associated with crossovers are revealed in the absence of mismatch repair genes in *Saccharomyces cerevisiae*.. Genetics.

[56] Hunter N, Borts RH (1997). Mlh1 is unique among mismatch repair proteins in its ability to promote crossing-over during meiosis.. Genes Dev.

[57] Mancera E, Bourgon R, Huber W, Steinmetz LM (2011). Genome-wide survey of post-meiotic segregation during yeast recombination.. Genome Biol.

[58] Hunter N, Chambers SR, Louis EJ, Borts RH (1996). The mismatch repair system contributes to meiotic sterility in an interspecific yeast hybrid.. EMBO J.

[59] Goldfarb T, Lichten M (2010). Frequent and efficient use of the sister chromatid for DNA double-strand break repair during budding yeast meiosis.. PLoS Biol.

[60] Cherry JM, Ball C, Weng S, Juvik G, Schmidt R (1997). Genetic and physical maps of *Saccharomyces cerevisiae*.. Nature.

[61] Buhler C, Borde V, Lichten M (2007). Mapping meiotic single-strand DNA reveals a new landscape of DNA double-strand breaks in *Saccharomyces cerevisiae*.. PLoS Biol.

[62] Pan J, Sasaki M, Kniewel R, Murakami H, Blitzblau HG (2011). A hierarchical combination of factors shapes the genome-wide topography of yeast meiotic recombination initiation.. Cell.

[63] Alani E, Reenan RA, Kolodner RD (1994). Interaction between mismatch repair and genetic recombination in *Saccharomyces cerevisiae*.. Genetics.

[64] Heck JA, Argueso JL, Gemici Z, Reeves RG, Bernard A (2006). Negative epistasis between natural variants of the *Saccharomyces cerevisiae MLH1* and *PMS1* genes results in a defect in mismatch repair.. Proc Natl Acad Sci U S A.

[65] Radford SJ, Sabourin MM, McMahan S, Sekelsky J (2007). Meiotic recombination in *Drosophila* Msh6 mutants yields discontinuous gene conversion tracts.. Genetics.

[66] Coic E, Gluck L, Fabre F (2000). Evidence for short-patch mismatch repair in *Saccharomyces cerevisiae*.. EMBO J.

[67] Foss HM, Hillers KJ, Stahl FW (1999). The conversion gradient at *HIS4* of *Saccharomyces cerevisiae*. II. A role for mismatch repair directed by biased resolution of the recombinational intermediate.. Genetics.

[68] Yeadon PJ, Rasmussen JP, Catcheside DE (2001). Recombination events in *Neurospora crassa* may cross a translocation breakpoint by a template-switching mechanism.. Genetics.

[69] Smith CE, Llorente B, Symington LS (2007). Template switching during break-induced replication.. Nature.

[70] Radford SJ, McMahan S, Blanton HL, Sekelsky J (2007). Heteroduplex DNA in meiotic recombination in Drosophila *mei-9* mutants.. Genetics.

[71] Kim KP, Weiner BM, Zhang L, Jordan A, Dekker J (2010). Sister cohesion and structural axis components mediate homolog bias of meiotic recombination.. Cell.

[72] Hunter N, Topics in Current Genetics, Molecular Genetics of Recombination, Aguilera A, Rothstein R (2007). Meiotic Recombination..

[73] Jessop L, Rockmill B, Roeder GS, Lichten M (2006). Meiotic chromosome synapsis-promoting proteins antagonize the anti-crossover activity of Sgs1.. PLoS Genet.

[74] Ira G, Malkova A, Liberi G, Foiani M, Haber JE (2003). Srs2 and Sgs1-Top3 suppress crossovers during double-strand break repair in yeast.. Cell.

[75] Rockmill B, Fung JC, Branda SS, Roeder GS (2003). The Sgs1 helicase regulates chromosome synapsis and meiotic crossing over.. Curr Biol.

[76] Oh SD, Lao JP, Hwang PY, Taylor AF, Smith GR (2007). BLM ortholog, Sgs1, prevents aberrant crossing-over by suppressing formation of multichromatid joint molecules.. Cell.

[77] Oh SD, Lao JP, Taylor AF, Smith GR, Hunter N (2008). RecQ helicase, Sgs1, and XPF family endonuclease, Mus81-Mms4, resolve aberrant joint molecules during meiotic recombination.. Mol Cell.

[78] Chelysheva L, Vezon D, Belcram K, Gendrot G, Grelon M (2008). The *Arabidopsis* BLAP75/Rmi1 homologue plays crucial roles in meiotic double-strand break repair.. PLoS Genet.

[79] Hartung F, Suer S, Knoll A, Wurz-Wildersinn R, Puchta H (2008). Topoisomerase 3alpha and RMI1 suppress somatic crossovers and are essential for resolution of meiotic recombination intermediates in *Arabidopsis thaliana*.. PLoS Genet.

[80] White CI (2008). News from *Arabidopsis* on the meiotic roles of Blap75/Rmi1 and Top3alpha.. PLoS Genet.

[81] Jessop L, Allers T, Lichten M (2005). Infrequent co-conversion of markers flanking a meiotic recombination initiation site in *Saccharomyces cerevisiae*.. Genetics.

[82] Ferguson DO, Holloman WK (1996). Recombinational repair of gaps in DNA is asymmetric in *Ustilago maydis* and can be explained by a migrating D-loop model.. Proc Natl Acad Sci U S A.

[83] Savage EA, Hastings PJ (1981). Marker effects and the nature of the recombination event at the *his 1* locus of *Saccharomices cerevisiae*.. Current Genetics.

[84] Bell LR, Byers B (1983). Homologous association of chromosomal DNA during yeast meiosis.. Cold Spring Harb Symp Quant Biol 47 Pt.

[85] Schwartz EK, Heyer WD (2011). Processing of joint molecule intermediates by structure-selective endonucleases during homologous recombination in eukaryotes.. Chromosoma.

[86] Schwacha A, Kleckner N (1995). Identification of double Holliday junctions as intermediates in meiotic recombination.. Cell.

[87] Evans E, Sugawara N, Haber JE, Alani E (2000). The *Saccharomyces cerevisiae* Msh2 mismatch repair protein localizes to recombination intermediates in vivo.. Mol Cell.

[88] Mortimer RK, Johnston JR (1986). Genealogy of principal strains of the yeast genetic stock center.. Genetics.

[89] Kane SM, Roth R (1974). Carbohydrate metabolism during ascospore development in yeast.. J Bacteriol.

[90] Baudin A, Ozier-Kalogeropoulos O, Denouel A, Lacroute F, Cullin C (1993). A simple and efficient method for direct gene deletion in *Saccharomyces cerevisiae*.. Nucleic Acids Res.

[91] Brudno M, Do CB, Cooper GM, Kim MF, Davydov E (2003). LAGAN and Multi-LAGAN: efficient tools for large-scale multiple alignment of genomic DNA.. Genome Res.

