# Supplementary material for: Genome-Wide Analysis of Heteroduplex DNA in Mismatch Repair–Deficient Yeast Cells Reveals Novel Properties of Meiotic Recombination Pathways
Source: PLoS Genet. 2011 Sep 29;7(9):e1002305. doi: 10.1371/journal.pgen.1002305 (PMC3183076; doi:10.1371/journal.pgen.1002305)
Supplement: Table S1 — List of strains. (DOC) [file pgen.1002305.s005.doc]

| **Table S1.** List of strains | | | |
| --- | --- | --- | --- |
| **strain number** | **genetic background** | **relevant genotype** | **origin** |
| NHY113 | S288C | MAT alpha, ade8 | N. Hunter |
| SKY1465 | SK1 | MAT alpha, ho::LYS2, ura3, leu2::hisG, lys2, arg4-Nsp::NdeI-site2^0^, thr1-A | Martini et al. 2006 |
| SKY1708 | SK1 | MAT a, ho::LYS2, ura3, leu2::hisG, lys2, arg4-Bgl::NdeI-site1^0^, CEN8::URA3 | Martini et al. 2006 |
| BLY107 | S288C | As NHY113 except *msh2*::HPH | This work |
| BLY109 | SK1 | As SKY1708 except *msh2*::HPH | This work |
| BLY114 | SK1 | BLY109 x SKY1465 ascospore, *msh2*::HPH | This work |
