# Supplementary material for: Genome-Wide Analysis of Heteroduplex DNA in Mismatch Repair–Deficient Yeast Cells Reveals Novel Properties of Meiotic Recombination Pathways
Source: PLoS Genet. 2011 Sep 29;7(9):e1002305. doi: 10.1371/journal.pgen.1002305 (PMC3183076; doi:10.1371/journal.pgen.1002305)
Supplement: Table S2 — Sequences of the oligonucleotides used to disrupt MSH2. (DOC) [file pgen.1002305.s006.doc]

| **Table S2:** Sequences of the oligonucleotides used to disrupt *MSH2* |
| --- |
| **CTTTATCTGCTGACCTAACATCAAAATCCTCAGATTAAAAGTATG**CGGATCCCCGGGTTAATTAA |
| **ATTATCTATCGATTCTCACTTAAGATGTCGTTGTAATATTAATTA**GAATTCGAGCTCGTTTAAAC |
| The *MSH2* targeted sequence is in bold. |
