# Supplementary material for: Genome-Wide Analysis of Heteroduplex DNA in Mismatch Repair–Deficient Yeast Cells Reveals Novel Properties of Meiotic Recombination Pathways
Source: PLoS Genet. 2011 Sep 29;7(9):e1002305. doi: 10.1371/journal.pgen.1002305 (PMC3183076; doi:10.1371/journal.pgen.1002305)
Supplement: Table S3 — List of array hybridizations. (DOC) [file pgen.1002305.s007.doc]

| **Table S3.** List of array hybridizations |  |  |
| --- | --- | --- |
| identifier | genotype | paper nomenclature |
| EX08_ADN0001_s1h1_Scerevisiae.CEL | s288c | NHY113_1 |
| Yeast_9_14-12-09_(Sc03b_MR_v04).CEL | s288c | NHY113_2 |
| Yeast_113_3.CEL | s288c | NHY113_3 |
| EX08_ADN0002_s1h1_Scerevisiae.CEL | SK1 | SKY1708_1 |
| Yeast_10_14-12-09_(Sc03b_MR_v04).CEL | SK1 | SKY1712_2 |
| Yeast_1712_3bis.CEL | SK1 | SKY1712_3 |
| Yeast_WT_meiose2_5.CEL | NA | WT1_A |
| Yeast_WT_meiose2_6.CEL | NA | WT1_B |
| Yeast_WT_meiose2_7.CEL | NA | WT1_C |
| Yeast_WT_meiose2_8.CEL | NA | WT1_D |
| Yeast_WT_meiose3_9.CEL | NA | WT2_A |
| Yeast_WT_meiose3_10.CEL | NA | WT2_B |
| Yeast_WT_meiose3_11.CEL | NA | WT2_C |
| Yeast_WT_meiose3_12.CEL | NA | WT2_D |
| Yeast_tetrade1_3.CEL | NA | WT3_A |
| Yeast_tetrade1_7.CEL | NA | WT3_B |
| Yeast_tetrade1_11.CEL | NA | WT3_C |
| Yeast_tetrade1_15.CEL | NA | WT3_D |
| Yeast_tetrade2_20.CEL | NA | WT4_A |
| Yeast_tetrade2_23-2.CEL | NA | WT4_B |
| Yeast_tetrade2_27-2.CEL | NA | WT4_C |
| Yeast_tetrade2_31-2.CEL | NA | WT4_D |
| EX08_ARN00015_s1h1_Scerevisiae.CEL | NA | WT5_A |
| EX08_ARN00016_s1h1_Scerevisiae.CEL | NA | WT5_B |
| EX08_ARN00017_s1h1_Scerevisiae.CEL | NA | WT5_C |
| EX08_ARN00018_s1h1_Scerevisiae.CEL | NA | WT5_D |
| EX08_ARN00019_s1h1_Scerevisiae.CEL | NA | WT6_A |
| EX08_ARN00020_s1h1_Scerevisiae.CEL | NA | WT6_B |
| EX08_ARN00021_s1h1_Scerevisiae.CEL | NA | WT6_C |
| EX08_ARN00022_s1h1_Scerevisiae.CEL | NA | WT6_D |
| EX08_ARN00023_s1h1_Scerevisiae.CEL | NA | WT7_A |
| EX08_ARN00024_s1h1_Scerevisiae.CEL | NA | WT7_B |
| EX08_ARN00025_s1h1_Scerevisiae.CEL | NA | WT7_C |
| EX08_ARN00026_s1h1_Scerevisiae.CEL | NA | WT7_D |
| Yeast_msh2_meiose1-spore1.CEL | NA | msh2_1_A1 |
| Yeast_msh2_meiose1-spore2.CEL | NA | msh2_1_A2 |
| Yeast_msh2_meiose1-spore3.CEL | NA | msh2_1_B1 |
| Yeast_msh2_meiose1-spore4.CEL | NA | msh2_1_B2 |
| Yeast_msh2_meiose1-spore5.CEL | NA | msh2_1_C1 |
| Yeast_msh2_meiose1-spore6.CEL | NA | msh2_1_C2 |
| Yeast_msh2_meiose1-spore7.CEL | NA | msh2_1_D1 |
| Yeast_msh2_meiose1-spore8.CEL | NA | msh2_1_D2 |
| Yeast_msh2_meiose2_2.CEL | NA | msh2_2_A |
| Yeast_msh2_meiose2_4.CEL | NA | msh2_2_B |
| Yeast_msh2_meiose2_6.CEL | NA | msh2_2_C |
| msh2_meiose2/Yeast_msh2_meiose2_8.CEL | NA | msh2_2_D |
| Yeast_1_14-12-09_(Sc03b_MR_v04).CEL | NA | msh2_3_A1 |
| Yeast_2_14-12-09_(Sc03b_MR_v04).CEL | NA | msh2_3_A2 |
| Yeast_3_14-12-09_(Sc03b_MR_v04).CEL | NA | msh2_3_B1 |
| Yeast_4_14-12-09_(Sc03b_MR_v04).CEL | NA | msh2_3_B2 |
| Yeast_5_14-12-09_(Sc03b_MR_v04).CEL | NA | msh2_3_C1 |
| Yeast_6_14-12-09_(Sc03b_MR_v04).CEL | NA | msh2_3_C2 |
| Yeast_7_14-12-09_(Sc03b_MR_v04).CEL | NA | msh2_3_D1 |
| Yeast_8_14-12-09_(Sc03b_MR_v04).CEL | NA | msh2_3_D2 |
