# Supplementary material for: Genome-Wide Analysis of Heteroduplex DNA in Mismatch Repair–Deficient Yeast Cells Reveals Novel Properties of Meiotic Recombination Pathways
Source: PLoS Genet. 2011 Sep 29;7(9):e1002305. doi: 10.1371/journal.pgen.1002305 (PMC3183076; doi:10.1371/journal.pgen.1002305)
Supplement: Table S5 — One marker based events. (DOC) [file pgen.1002305.s009.doc]

| **Table S5**: One marker based events | | | |
| --- | --- | --- | --- |
| tetrad | S288C converted marker | SK1 converted marker | total |
| WT1 | 35 | 4 | 39 |
| WT2 | 5 | 0 | 5 |
| WT3 | 9 | 0 | 9 |
| WT4 | 30 | 6 | 36 |
| WT5 | 2 | 1 | 3 |
| WT6 | 6 | 3 | 9 |
| WT7 | 4 | 4 | 8 |
| msh2_1 | 18 | 29 | 47 |
| msh2_3 | 16 | 8 | 24 |
